# Ameloblastic Carcinoma in a 2-Year-Old Child: A Case Report and Review of the Literature

**DOI:** 10.1155/2020/4072890

**Published:** 2020-07-22

**Authors:** Ngoc Bao Vu, Ngoc Tuyen Le, Risa Chaisuparat, Pasutha Thunyakitpisal, Ngoc Minh Tran

**Affiliations:** ^1^Dental Biomaterials Science Program, Graduate School, Chulalongkorn University, Bangkok 10330, Thailand; ^2^Department of Maxillofacial and Plastic Surgery, Hanoi National Hospital of Odonto-Stomatology, Hanoi 100000, Vietnam; ^3^Department of Maxillofacial Reconstructive Surgery, Hanoi National Hospital of Odonto-Stomatology, Hanoi 100000, Vietnam; ^4^Department of Oral Pathology, Faculty of Dentistry, Chulalongkorn University, Bangkok 10330, Thailand; ^5^Research Unit of Herbal Medicine, Biomaterial, And Material for Dental Treatment, Department of Anatomy, Faculty of Dentistry, Chulalongkorn University, Bangkok 10330, Thailand; ^6^Department of Pathology, Hanoi Medical University, Hanoi 100000, Vietnam

## Abstract

Ameloblastic carcinoma (AC) is a rare malignant odontogenic tumor in pediatric patients, only 22 cases have been reported in literature since 1932. We present an extremely rare case in which AC occurred in a 2-year-old girl, who had a tumor in the right mandible. Radiographic findings showed a multilocular, poorly defined, and mixed radiolucent-radiopaque lesion in the region of teeth #84 to #85, with bone and tooth root resorption. Computed tomography revealed buccal cortex destruction, tumor infiltration of soft tissue, and enlarged nodes. Incisional biopsy showed histomorphological features of AC. Immunohistochemical analysis exhibited a positive result for Cytokeratin (CK) 19 and overexpression of p53 and Ki67. The patient underwent right hemimandibulectomy and neck dissection. The final pathology was consistent with the initial diagnosis of AC. The patient did not exhibit signs of recurrence or metastasis within 2 years postoperatively. Given the rarity of this disease and the age of the patient, this report constitutes a valuable contribution to the current literature.

## 1. Introduction

Ameloblastic carcinoma, first described by Elzay in 1982, is a rare, malignant type of odontogenic tumor [1]. AC has features of both ameloblastoma and carcinoma, independent of the presence of metastasis; it should be distinguished from malignant (metastasizing) ameloblastoma (MA), which exhibits benign histological appearance of ameloblastoma in primary and metastatic lesions [2]. In 2005, the World Health Organization classification of odontogenic tumors included AC as a malignant tumor, in a manner similar to that for MA. However, in the most recent the World Health Organization classification (2017), MA was reclassified as a benign odontogenic tumor, whereas AC continues to be considered a rare and highly malignant odontogenic tumor [3–5].

AC involves the mandible more frequently than the maxilla. It most frequently affects adult men. However, a few pediatric cases have been reported, with a minimum age of 4 [6, 7]. In this report, we describe a 2-year-old girl who was diagnosed with right mandibular ameloblastic carcinoma.

## 2. Case Description

Written informed consent of the patient's mother was obtained prior to this paper's publication.

A 2-year-old girl presented with a painful mass in the right mandible, which had appeared 1 month prior. Her first examination was performed in a local hospital, and the initial diagnosis was gingivitis. Oral hygiene instruction and antibiotics were prescribed. One week later, the mass continued to increase in size and caused pain and fever; thus, the girl was admitted to the Department of Maxillofacial Surgery at Hanoi National Hospital of Odonto-Stomatology, Hanoi, Vietnam.

Clinical examination revealed a mass in the right body of the mandible that extended from the commissure of the lip to the angle of the mandible. It was approximately 1 × 1 cm in size and painful on palpation. No sign of lip paresthesia related to the mass was detected, and the overlying skin was normal in color and texture. The submandibular lymph nodes were palpable, tender but painless, and movable. Mouth opening was normal. Intraoral examination showed a swelling in the region of teeth #83 to #85, which obliterated the right buccal sulcus. Mobility and displacement of teeth #84 and #85 were detected. The lesion was soft in consistency and painful on intraoral palpation. The overlying mucosa exhibited overgrowth (covering the crown of #85), red color, and an ulcerated appearance.

### 2.1. Radiographic Findings

**Figure 1:**
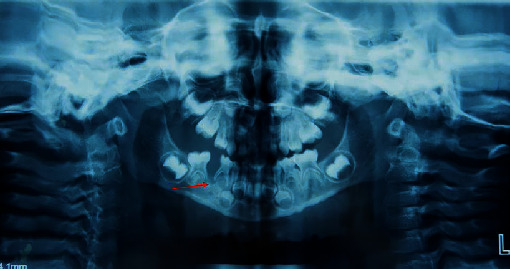
Orthopantomograph showing the presence of an ill-defined, mixed radiolucent-radiopaque lesion involving the #84 to #85 region. Bone and roots resorption can be observed (red arrow).

**Figure 2:**
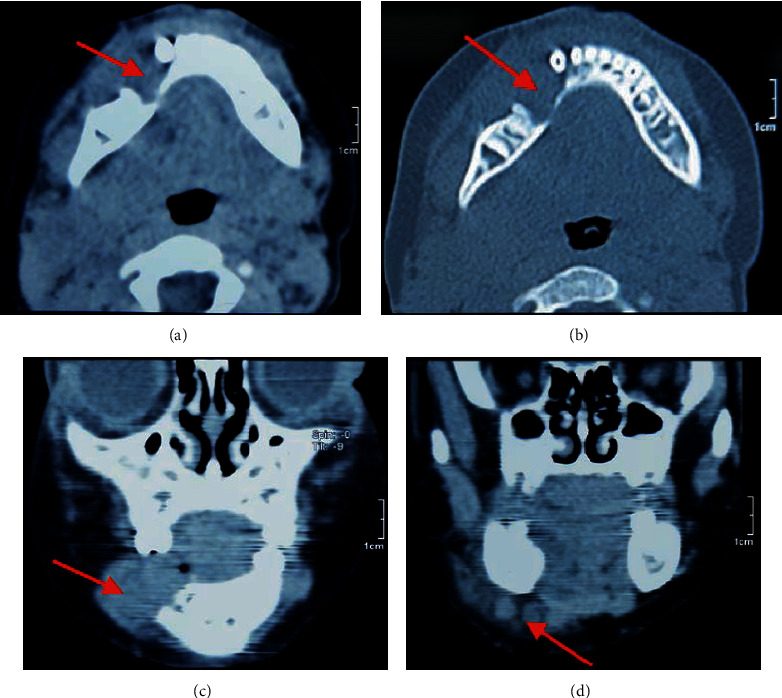
CT images showing a lesion with bony cortex perforation (red arrow, (a) and (b)) and soft tissue infiltration and lymph nodes involvement (red arrow, (c) and (d)).

Orthopantomography showed a multilocular, mixed radiolucent, and radiopaque lesion with a poorly defined border affecting the region of teeth #84 and #85. The lamina dura, roots of #84 and #85, and furcation of #84 were resorbed (Figure 1). Axial and coronal computed tomography revealed a poorly defined lesion in the right body of the mandible. The tumor had destroyed the buccal cortex and infiltrated into the soft tissue; reactive lymph nodes may be observed. The lingual cortex was partially damaged (Figure 2). Computed tomography of the chest revealed no metastatic deposits.

### 2.2. Biopsy and Histological Findings

**Figure 3:**
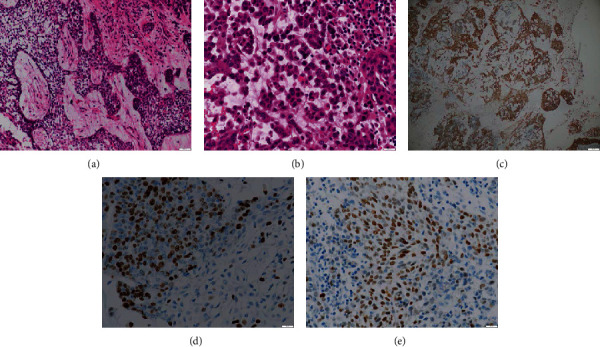
Photomicrographs showing sheets and nests of ameloblastic epithelium with inflammatory infiltration ((a) hematoxylin eosin); areas of tumor cells with hyperchromatism, nuclei pleomorphism, and some mitotic figures ((b) hematoxylin eosin); marked positive immunohistochemical expression of CK19 (c), Ki67 (d), and p53 (e).

Incisional biopsy was performed at the intraoral vestibule, where the lesion had penetrated. Histologic examination revealed sheets and nests of odontogenic epithelium separated by fibrous tissue with inflammatory infiltration and stellate reticulum-like structure. The peripheral cells of the nests resembled preameloblasts with cuboid shape and nuclei polarization (Figure 3(a)). Dedifferentiated areas exhibited cytologic malignant cells with increased nucleocytoplasmic ratio and hyperchromatic nuclei; few mitotic figures were present (Figure 3(b)). Histomorphological analysis demonstrated an aggressive type of ameloblastoma, suggestive of AC.

Immunohistochemical stains were performed using CK19, Ki67, and p53. The ameloblastic epithelium showed a positive reactivity for CK19 (Figure 3(c)). High proliferation level of the neoplasm was confirmed by elevated expression of Ki67 and p53 (Figures 3(d) and 3(e)).

Based on these findings, the final diagnosis was ameloblastic carcinoma.

### 2.3. Surgery

**Figure 4:**
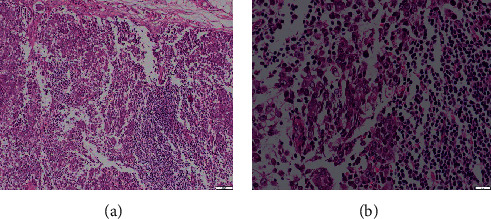
Photomicrographs showing tumor infiltration in the submandibular lymph node: dedifferentiated area with infiltrated neoplastic cells ((a) hematoxylin eosin); higher power view of the infiltrated lymph node showing nuclear pleomorphism and hyperchromatism of the neoplastic cells ((b) hematoxylin eosin).

The patient underwent right hemimandibulectomy, extending from the distal aspect of #71 to the angle of the right mandible, with safe osseous margins of 2 cm on each side of the tumor. The surrounding tissue was also excised. Complete supraomohyoid neck dissection was performed on the right side, combined with excision of the right submandibular gland. The patient recovered uneventfully, and the wound healed well after surgery. The histopathological examination of the resection specimens was performed, and the diagnosis of the primary tumor was consistent with the initial diagnosis of AC. The positive submandibular lymph nodes were identified, and the microscopic examination of the lymph nodes showed diffuse infiltration of neoplastic cells (Figure 4). The submandibular salivary gland was not involved (data not shown).

### 2.4. Follow-Up

**Figure 5:**
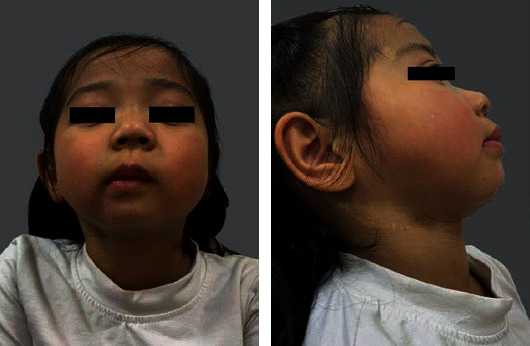
Photographs of the patient at 2-year postoperation.

The patient returned regularly for follow-up. Clinical and radiographic examination at 2 years postoperatively showed no sign of recurrence or metastasis (Figure 5).

## 3. Discussion

AC is considered a rare, malignant neoplasm of odontogenic origin. Patients of various ages can be affected, but the disease most commonly occurs during the fourth decade of life. Moreover, it appears more frequently in men than in women, and the mandible is affected more frequently than the maxilla [8].

AC shares some common clinical features with ameloblastoma, such as a mass in the jaw, resorption of bone, and mobility of teeth. However, its behavior is more aggressive, including rapid growth, pain, perforation of the cortical plate, soft tissue infiltration, and/or lower lip paresthesia [9]. Lymph node involvement has been reported as a dominant sign of metastasis [10]. The radiographic features of AC are comparable to those of ameloblastoma: unilocular or multilocular radiolucent lesions with lamina dura and tooth apex resorption. However, AC may exhibit focal radiopacities, dystrophic calcification, and a poorly defined lesion border [11].

Histologic features, such as cytologic atypia and increased mitotic figures, are important criteria for distinguishing AC from ameloblastoma [12]. When assessing carcinoma in the jaw, it is first necessary to exclude the metastasis or invasion of bone by neoplasm from adjacent tissue or the paranasal sinus, as well as metastasis in the jaw from visceral tumors [13]. The first consideration in differential diagnosis of AC is primary intraosseous carcinoma. In addition to epidemiologic and clinical differences, histological features of primary intraosseous carcinoma compared to AC include less differentiation and a lack of keratinization. Squamous cell carcinoma arising in the lining of an odontogenic cyst is another potential differential diagnosis, but its histological appearance more closely resembles that of oral squamous cell carcinoma [10].

Some immunohistochemical markers correlating with the diagnosis of AC have been identified. In our report, immunohistochemistry is employed for interpreting the tumor origin and biological behaviors. CK19 expression is detected in the epithelium of the dental germ, so it has been a good marker for odontogenic cysts and tumors, such as ameloblastoma. Ki67 is a nuclear protein which is presented in cellular proliferation. The immunoexpression of Ki67 has been considered as a prognostic tool to distinguish among benign and malignant tumor. p53, known as tumor suppressor gene, plays an important role in DNA repair and apoptosis initiation. The accumulation of p53 has been associated with increased cellular proliferation and malignant transformation. In Martínez et al.'s study comparing histological and immunohistochemical features of ameloblastoma and AC, they suggested that both Ki67 and p53 could be good markers of malignancy [14].

Little information regarding AC in pediatric patients is available. A review of the literature from 1932 to 2019 revealed 22 cases in pediatric patients, for whom age, sex, location, clinical signs, treatment, follow-up, recurrence, and metastasis status were collected [6]. However, a few details were unavailable (Table 1). Patient age ranged from 4 to 17 years, with a mean of 12.98 years; the male-to-female ratio was 3 : 1. In total, 64% of the patients had AC in the mandible. Swelling was the first symptom in 64% of the patients; other signs included pain, dysphonia, and trismus. Surgery was performed in 17 of 22 patients; three patients were treated with both surgery and radiotherapy because of the involvement of surgical margins. Chemotherapy alone was administered in one patient. Treatment details were not clear in 4 patients before 1979. The follow-up duration ranged from 0.5 to 24 years (mean of 6.4 years). In the review, we noted that recurrence occurred after an extended interval, from 1 to 16.3 years posttreatment, and was detected in 24% of the patients. Metastasis was detected in eight patients (1 bone, 1 lymph node, 1 lung, and 5 patients with multiple metastases). Six of the 22 patients (27.3%) were reported to have died; four of these had metastasis.

The management of AC remains controversial, but surgical resection is always recommended. En bloc removal of the jaw with 2 cm of normal bone margin is indicated to assure a disease-free status. This approach results in a recurrence rate below 15% [15]. Cervical dissection should be considered when there is a sign of lymph node metastasis. Postoperative radiotherapy and chemotherapy may be useful; however, the outcomes of these therapies have not been well documented [16].

This report appears to describe the youngest patient with AC reported in the literature to date. The child had a rapidly growing and aggressive lesion, with bone destruction and suspected lymph node metastasis. The diagnosis was based on histopathologic and immunohistochemical features, including ameloblastic differentiation, nuclear pleomorphism, mitotic figures, and positive reactivities with specific immunomarkers. The treatments were segmental mandibulectomy (with safe bony margins of 2 cm) and neck dissection. No adjuvant therapy was applied. Regular check-ups were performed every 3 months, and the long-term prognosis for this patient is expected to be good.

## 4. Conclusion

To date, AC is still a rare, highly malignant odontogenic tumor, with a 5-year survival rate below 70%. Metastases to the lung, liver, lymph nodes, bone, and brain are the causes of death and may appear at 0.5 to 14 years postoperatively. Thus, radical treatment and meticulous long-term follow-up are essential, and sufficient time should be considered before reconstruction due to the potential for tumor recurrence.

## Figures and Tables

**Table 1:** Published pediatric cases of AC (1932 to 2019).

Case	Authors	Age (y)	Gender	Site	Initial signs	Treatment	Follow-up (mo)	Metastasis/recurrence	Dead/alive
1	Spring (1932) [17]	5	Male	Mandible	Not mentioned	Not mentioned	168	Bone	Dead
2	Villa (1958) [18]	17	Male	Mandible	Swelling	Not mentioned	0		Alive
3	Herceg and Harding (1972) [19]	9	Male	Mandible	Not mentioned	Not mentioned	121	Lung+liver+lymph node	Dead
4	Höltje and Donath (1977) [7]	4	Male	Mandible	Not mentioned	Not mentioned	36		Dead
5	Krempien et al. (1979) [20]	5.5	Male	Maxilla	Swelling	Surgery	144	Lung	Alive
6	Nadimi et al. (1986) [21]	15	Female	Maxilla	Not mentioned	Surgery	0		Alive
7	Corio et al. (1987) [10]	15	Male	Maxilla	Swelling	Surgery	12		Alive
8	Corio et al. (1987) [10]	17	Male	Mandible	Pain, swelling, dysphonia	Surgery	12	Recurrence	Alive
9	Hall et al. (2007) [22]	15	Male	Maxilla	Swelling	Surgery	196	Recurrence	Alive
10	Hall et al. (2007) [22]	16	Male	Maxilla	Swelling	Surgery	288		Alive
11	Hall et al. (2007) [22]	7	Female	Maxilla	Swelling	Surgery	119	Recurrence	Alive
12	Hall et al. (2007) [22]	17	Female	Mandible	Swelling	Surgery	122	Recurrence	Dead
13	Yazici et al. (2008) [23]	10	Male	Maxilla	Swelling	Surgery+radiotherapy	6		Alive
14	Reid-Nicholson et al. (2009) [24]	15	Male	Mandible	Swelling	Surgery	Not mentioned	Lymph node	Not mentioned
15	Cherry et al. (2009) [25]	16	Male	Mandible	Swelling, pain	Surgery+radiotherapy	Not mentioned	Brain+lung	Alive
16	Ndukwe et al. (2010) [26]	16	Male	Mandible	Not mentioned	Surgery	Not mentioned		Not mentioned
17	Ndukwe et al. (2010) [26]	16	Female	Mandible	Not mentioned	Surgery	Not mentioned		Not mentioned
18	Devenney-Cakir et al. (2010) [27]	16	Male	Mandible	Swelling, trismus	Surgery+radiotherapy	48	Brain+lung	Alive
19	Horváth et al. (2012) [28]	8	Female	Mandible	Pain	Chemotherapy	8	Lung+bone	Dead
20	Yoshioka et al. (2013) [29]	17	Male	Mandible	Swelling	Surgery	39	Lung+recurrence	Dead
21	Sozzi et al. (2014) [6]	14	Male	Maxilla	Swelling	Surgery	24		Alive
22	Fahradyan et al. (2019) [30]	15	Male	Mandible	Swelling	Surgery	30		Alive
